# Bone and Joint Infections among Hematopoietic Stem Cell Transplant Recipients

**DOI:** 10.7150/jbji.38120

**Published:** 2019-09-18

**Authors:** Cybele Lara Abad, Vania Phuoc, Prashant Kapoor, Pritish K. Tosh, Irene G. Sia, Douglas R. Osmon, Aaron J. Tande

**Affiliations:** 1 Department of Internal Medicine, Section of Infectious Diseases, University of the Philippines-Manila, Philippine General Hospital, Manila Philippines;; 2 Division of Hematology, University of the Philippines-Manila, Philippine General Hospital, Manila Philippines;; 3 Division of Infectious Diseases, University of the Philippines-Manila, Philippine General Hospital, Manila Philippines;; 4 Mayo Clinic, Rochester MN, USA.

**Keywords:** Hematopoietic stem cell transplantation, bone, joint

## Abstract

**Background:** Hematopoietic stem cell transplantation (HSCT) recipients are at increased risk for infection. This study describes bone and joint infections (BJI) among HSCT recipients.

**Methods:** We reviewed 5861 patients who underwent HSCT at Mayo Clinic, Rochester, MN from January 1, 2005 through January 1, 2015 for study inclusion. BJI was defined as native septic arthritis, prosthetic joint infection, osteomyelitis, and orthopedic implant infection. All adults with BJI after HSCT were included in the analysis.

**Results:** Of 5861 patients, 33 (0.6%) developed BJI. Native joint septic arthritis was the most common BJI occurring in 15/33 (45.4%) patients. Patients were predominantly male (24/33, 72.7%), with median age of 58 (range 20-72) years. BJI was diagnosed a median of 39 (range 1-114) months after allogeneic (14/33, 42.4%) or autologous (19/33, 57.6%) HSCT. Organisms were recovered via tissue (24/27, 88.9%), synovial fluid (13/17, 76.5%), and/or blood cultures (16/25, 64%). Most underwent surgical debridement (23/33, 69.7%). Patients were followed a median of 78.3 months (range 74-119). Therapy was unsuccessful in 4/33 (12.1%), with death related to the underlying BJI in two (50%). Failure occurred a median of 3.4 (0.1-48.5) months from diagnosis. At last follow up, 7/33 (21.2%) patients were alive. Median overall survival was 13 months (0.07-70.6).

**Conclusion:** BJI among HSCT recipients is infrequent. The most common infection is native joint septic arthritis. Pathogens appear similar to patients without HSCT. Treatment involving surgical-medical modalities is successful, with most patients surviving >1 year after BJI.

## Introduction

Hematopoietic stem cell transplant (HSCT) recipients are at high risk for infections caused by common and opportunistic pathogens. The prolonged state of severe neutropenia in the pre-engraftment period, the depletion of major cell lines after transplant, and the occurrence of acute graft versus host disease for allogeneic transplants increase the net state of immune suppression for a prolonged period of time. It is not surprising that infection is the leading cause of morbidity and mortality among HSCT recipients. Presently, information regarding bone and joint infections (BJIs) among HSCT recipients is limited to case reports 1-4. We therefore retrospectively reviewed cases of BJI among HSCT recipients at Mayo Clinic to describe the epidemiology, clinical features, microbiology, treatment, and outcome of infection.

## Methods

### Patient Selection

All patients who underwent HSCT at Mayo Clinic, Rochester, from January 1, 2005 - January 1, 2015 were eligible for study inclusion. By using International Classification of Disease (ICD) codes, histopathology results, and Advanced Cohort Explorer (ACE), a computerized medical record search engine, we identified 107 patients with potential BJI and reviewed their electronic medical record (EMR) for occurrence of BJI. Microbiology data was searched only after BJI was confirmed through the EMR. As per Minnesota statute, only those who provided informed consent to allow review of their medical records for research purposes were included. The Mayo Clinic Institutional Review Board approved the study. Pediatric recipients <18 years old, those who developed a BJI prior to transplantation, those with head and neck (e.g. mandibular) infection, or those who died on the day of transplantation were excluded from the study.

### Definitions

We followed established criteria to define BJIs 5,6: *Vertebral and non-vertebral (e.g. appendicular) osteomyelitis* was defined as pain or purulence around the affected site with proof of bony involvement via radiographic imaging, with either growth of organism via bone biopsy or histopathologic findings consistent with acute or chronic osteomyelitis. Criteria for *prosthetic joint infection* included 2 or more of the following: 1) the presence of a sinus tract communicating with the prosthesis; 2) the presence of acute inflammation seen on histopathologic examination of periprosthetic tissue at the time of surgical debridement or prosthesis removal; 3) the presence of purulence without another known etiology surrounding the prosthesis or 4) two or more intraoperative cultures or combination of preoperative aspiration and intraoperative cultures that yield the same organism. *Native joint septic arthritis* was defined as infection of the joint space as evidenced by synovial fluid analysis and/or the presence of inflammation or edema (around the joint space) seen via imaging, with or without the isolation of organism/s from the joint itself. *Orthopedic implant infection* was considered when pain and/or purulence developed around a metal implant or hardware.

Infections were classified according to the type of BJI - osteomyelitis, native joint septic arthritis, PJI - and/or according to anatomic site (e.g., spine, knee, hip). Treatment outcome was defined as a failure if there was either progression of infection, relapse, or reinfection.

### Statistical Analysis

Descriptive statistics on baseline variables were reported as frequency (percentage) for categorical variables and as quartiles (median, minimum and maximum) for continuous variables. For outcome variables (e.g. success vs. failure), cumulative event rates were estimated as a function of time during their last follow up after transplantation using the Kaplan-Meier product limit method. In this calculation, those who died were censored at the time of their last follow-up visit. Overall survival (OS) was defined as the interval from the time of diagnosis of the BJI until death or last follow-up. The patients who were alive at the time of last follow-up were censored for the calculation of OS.

## Results

### Patient Characteristics

**Figure 1:**
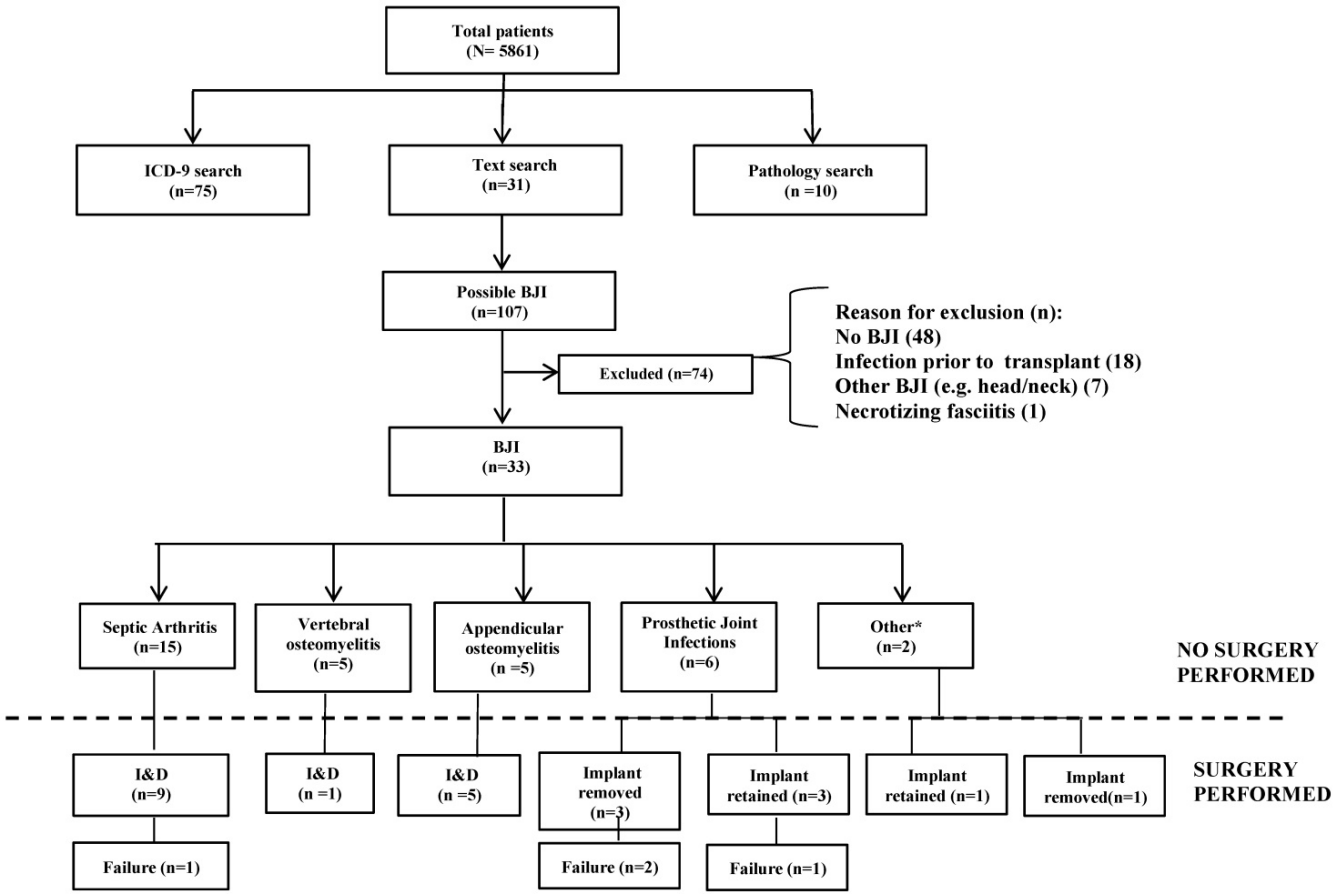
Flowchart of the Transplant study cohort. LEGEND: HSCT = Hematopoietic stem cell transplant BJI = Bone and Joint Infection *Other -orthopedic implant infection.

There were 5861 HSCT patients identified from the Mayo Clinic Transplant database within the study period. Of these, 33 (0.6%) developed BJI. A flow diagram of the study cohort is shown in Figure 1. Most patients were male (24/33, 72.7%) and median patient age was 58 (range 20-72) years old. An underlying lymphoid (24/33, 72.7%) or myeloid (9/33, 27.3%) malignancy was the reason for allogeneic (14/33, 42.4%) or autologous (19/33, 57.6%) HSCT. Most patients (24/33, 72.7%) had at least one co-morbid illness, including Type 2 diabetes mellitus (DM) (7/33, 21.2%), chronic kidney disease (CKD) (9/33, 27.3%), and autoimmune disease (4/33, 12.1%) (Table 1).

### Characteristics of Infection

#### Overall

BJI was diagnosed a median of 39 (range 1-114) months after HSCT. Median time to BJI diagnosis was 51 (range 6-114) and 13 (range 1-85) months for autologous and allogeneic HSCT recipients, respectively. At the time of BJI diagnosis, 24 (13 allogeneic, and 11 autologous) were considered cured or in remission.

Overall, native joint septic arthritis was the most common BJI occurring in 15/33 (45.4%) patients, followed by osteomyelitis (n = 10), prosthetic joint infections (n= 6), and orthopedic implant associated infection (n = 2) (Figure 1). The overall rates of septic arthritis, osteomyelitis, and prosthetic joint infection in the entire HSCT cohort (N=5861) were extremely low at 0.3, 0.2, and 0.1, respectively. Only 7 patients were neutropenic (median ANC 900/mm^3^) at time of diagnosis of infection. For majority of patients, both erythrocyte sedimentation rate (ESR) (24/28) and C-reactive protein (CRP) (27/27) were elevated, at a median of 46 (range 1-138) mm/h and 38.1 (range 4.8-346.4) mg/L, respectively. Blood cultures were performed in 25/33 patients, and 16 of these were positive (64%). Organisms were recovered via tissue culture in majority of cases (24/27). A summary of infections according to type of BJI is in Table 2.

#### Native Joint Septic Arthritis

Fifteen patients, 11 of whom were male (73.3%), developed native joint septic arthritis. The knee was the most common joint infected, occurring in 8 patients, followed by the hip joint in 3, and ankle (n=2) and shoulder (n =2). Median age and time to diagnosis was 51 (range 35-76) years and 51 (range 1-89) months, respectively. The underlying malignancy was a lymphoid disorder in 11/15 HSCT recipients, of which 8 were multiple myeloma. Median ESR and CRP were 54 mm/hr (range 1-137) and 72 mg/L (range 14.2-268.7), respectively. Synovial fluid analysis was performed in 13 of 15 patients and median cell count was elevated at 14,872 cells/µl. Positive synovial fluid (9/13, 69.2%), intraoperative tissue or fluid (9/9, 100%) and blood cultures (9/14, 64.3%) correlated well with each other. The organisms isolated from tissue were predominantly gram positive - *Staphylococcus aureus (*n = 1*), Streptococcus pneumoniae (*n = 2*), Enterococcus faecalis (*n = 1*),* coagulase negative* Staphylococcus* species (CoNS) (n = 1)*,* and *Streptococcus pyogenes* (n = 1). Other organisms included *Mycobacterium chelonae* (n = 1), *Pseudomonas aeruginosa* (n = 1), and polymicrobial *Klebsiella pneumoniae* and *Enterobacter* sp. infection (n = 1).

#### Osteomyelitis

Ten patients (8 male, 2 female) developed vertebral (n = 5) and appendicular (n = 5) osteomyelitis at a median age of 60 years (range 23-71). Appendicular osteomyelitis occurred in 3 of 5 patients with diabetes mellitus, of whom 2 had diabetic foot infections. Other sites included a left lower extremity stump site (1), scapula (1), and iliac crest (1). Median time to diagnosis was 30.5 months (range 1-114) after HSCT. The underlying malignancy was a lymphoid disorder in half of patients, and 4/10 (40%) received autologous HSCT. Magnetic resonance imaging (MRI) was the radiologic imaging modality in 4/10 patients, with others undergoing x-ray (1), Computed Tomography (CT) imaging (2), or Indium-labeled scan (2). One did not undergo radiographic imaging at Mayo Clinic. Median ESR and CRP were 42 mm/hr (4-62) and 26.1 mg/L (5.5-34.4), respectively. All patients tested (n=6) had elevated CRP, while ESR was normal in 1. The majority of bone biopsy cultures were positive (8/10, 80%), which correlated with blood cultures in 4 of 6 patients (66.7%). Infections were predominantly monomicrobial - *E. faecalis* (1), CoNS (2), *S. aureus* (1), *Peptostreptococcus* sp. (1), and *K*.*pneumoniae* (1). One patient had both *S.aureus* and *Peptostreptococcus* sp., and the other had multiple organisms.

#### Prosthetic Joint Infections

Six patients (18.2%) developed PJIs. These occurred at a median age of 65.5 years (range 49-72), among 4 males and 2 females. All patients had an underlying lymphoid malignancy. Four received autologous HSCT. Median time to PJI diagnosis was 38 months (range, 6-99) after HSCT. There were 3 hip, 2 knee, and 1 elbow prosthetic infection. X-ray was used as the imaging of choice for half of patients. Median ESR and CRP were both 51 (range 13-103 mm/hr and <3-346.4 mg/L, respectively). Synovial fluid analysis with culture was performed for 4/6 patients, with median cell count of 37,783 cells/mm^3^. Tissue cultures for all patients were positive - (3) CoNS, (2) *Staphylococcus aureus*, and (1) *Streptococcus pneumoniae*. Blood cultures were positive in 2/4 patients.

#### Other Infections

Two patients with underlying lymphoid malignancy developed orthopedic implant infection of external fixators at a median age of 52.5 years. The infection occurred a median of 24.5 months after transplantation. Median ESR and CRP were high at 80.5 mm/hr and 8.25 mg/dL, respectively. Tissue culture was negative in one, and showed *Cutibacterium acnes* (formerly *Propionibacterium acnes)* in another. Blood culture was documented only in one patient, and was negative.

### Treatment and Outcome

#### Overall

All patients were given induction antimicrobial therapy, lasting a median of 34 (range 3-168) days. The majority underwent concomitant surgical debridement (23/33, 69.7%). Eight patients (24.2%) were subsequently placed on suppressive therapy. Patients were followed a median of 6.7 months (range 0.1-121). Most patients were successfully treated (29/33, 87.9%) while 4 were considered treatment failures. Failure occurred a median of 3.4 (range 0.1-48.5) months from diagnosis and was due to either recurrence of symptoms (2/4) or persistently positive tissue cultures (2/4). At last follow up, 7/33 (21%) patients were alive, of whom 6 were treated successfully. Median overall survival was 13 months (0.07.-70.6). A summary of treatment according to type of infection is in Table 3.

#### Native Joint Septic Arthritis

The majority of patients received intravenous antimicrobials initially, with the exception of one patient who was given oral antimicrobial therapy. Monotherapy was preferred for most patients (9/15). Median length of induction therapy was 29 days (range 3-108). Four patients were transitioned to oral therapy, and 2 of these were placed on chronic indefinite suppressive therapy. In addition to intravenous antimicrobials, 9 patients underwent surgery - 4 had open arthrotomy, 4 had arthroscopic irrigation and debridement, one had a Girdlestone procedure. A minority (2/9, 22.2%) had a second surgery within a year of infection diagnosis. Treatment was successful with the exception of one patient, who developed septic shock from the underlying infection. On last follow up, only 5/15 (33.3%) were alive. The cause of death was progression of the underlying malignancy (5), followed by unknown cause (3), progression of the underlying joint infection (1) or a different infection (1).

#### Osteomyelitis

All patients were given intravenous antibiotic therapy, either alone (5/10), or in combination with another oral (2) or intravenous (3) agent. Median length of induction treatment was 42 (range18-62) days. Five patients were transitioned to oral treatment thereafter, with 4 placed on lifelong oral antimicrobials. All patients with appendicular osteomyelitis (5) and one patient with vertebral osteomyelitis had open surgery in addition to antibiotics. One patient had 3 surgical procedures. All patients were successfully treated. However, on last follow up, 4 had died from progression of the underlying malignancy, 4 of an unrelated infection, and one of an unknown cause.

#### Prosthetic Joint Infection

All patients with PJI were given intravenous antimicrobial therapy. The median duration of induction therapy was 47 (range 32-168) days. Half were given dual antibiotic treatment, 2 with oral rifampin, and 1 with moxifloxacin. Three patients were transitioned to oral therapy, with 2 others given lifelong oral medications. All patients had surgery - 3 patients underwent two-stage arthroplasty exchange, 2 had debridement and implant retention and polyethylene exchange, and one had debridement and implant retention without polyethylene exchange. One patient had multiple (8) surgical procedures. Only half of patients with PJI were successfully treated; the remainder had relapse (2) or re-infection (1). On last follow up, most (5/6) were deceased, from progression of the underlying malignancy, (2) unknown causes (2), or (1) from an unrelated infection.

#### Other Infections

The 2 patients with orthopedic implant infection were treated with surgery and intravenous antimicrobials. Median duration of treatment was 21.5 (14-29) days, before a prolonged maintenance phase on oral antimicrobials. Surgery involved one-stage implant exchange in one patient and irrigation and debridement with implant retention in the other. Both patients were treated successfully but later succumbed to an unrelated infection.

## Discussion

Our study is the first to describe BJIs among HSCT recipients. In this large cohort, we only identified 33 recipients who developed a BJI infection within the 10-year study period. In general, infections occurred late (median of 39 months), well beyond the period of profound neutropenia, which likely did not play a role in the pathogenesis of this type of infection. However, compared to autologous HSCT recipients, allogeneic HSCT recipients tended to present with BJI earlier (13 vs. 51 months), possibly related to the more profound state of immune suppression, although this was not statistically significant. Characteristics typical of BJI in the immunocompetent population, such as elevated inflammatory markers, positive synovial fluid or tissue cultures, and predilection for gram-positive organisms, were also commonly observed among HSCT recipients.

Native joint septic arthritis was the most common type of BJI occurring in 45.4% (15/33) of our cohort, over half (8/15) of whom had underlying multiple myeloma. The most common organisms identified were gram positive, which is frequently observed in immune competent hosts. The relatively higher frequency of arthritis from *S. pneumoniae* (2/15), however, could be related to the underlying hematologic malignancy. Among the few cases of septic arthritis reported in HSCT recipients 2-4,7-10, pneumococcal arthritis occurred in four 3,8-10. The pathogenesis is not fully ascertained, but we surmise that the underlying hypogamma-globulinemia present in these patients (e.g. multiple myeloma) plays a role in increasing the risk of infection with these encapsulated organisms. Reports of septic arthritis from organisms such as *Salmonella* 4, and *H. influenzae* 7 that also depend on adequate B-cell function, makes this theory plausible.

We found 10 cases of osteomyelitis in our cohort. All cases were bacterial in nature, and pathogens were predominantly gram positive, which is similar to the immune competent population^11^. Knowledge regarding osteomyelitis among HSCT recipients remains scant and is limited to case reports^12^-^15^. Based on these reports, there seems to be an increased risk for infection with atypical organisms such as non-tuberculous mycobacteria (NTM) or fungi among immune compromised hosts, but our study did not validate this association and suggests a publication bias in the previous reports.

Prosthetic joint infection developed in 6 of our HSCT recipients. To our knowledge, PJI among HSCT recipients has not yet been reported. In contrast, there is a large retrospective review of PJI among 367 solid organ transplant (SOT) recipients, of whom 12 developed PJI^16^. We did not collect the prevalence of joint arthroplasty in our cohort, so it is difficult to compare the relative incidence of PJI between SOT and HSCT populations. The causative agents for all HSCT recipients with PJI were gram positive, which closely mirrors the microbiology of the general population^17^ and the SOT population^16^. Half of the patients (3/6) had poor outcome with recurrence of infection despite removal of the infected arthroplasty in 2 of 3 patients. Not surprisingly, the patients with arthroplasty retention developed recurrent infection much earlier (11 vs. 822 days). A potential reason for failure could be the shorter duration of induction therapy in those that failed, versus those that were successfully treated (median 42 days vs. 80 days), but this difference was not statistically significant.

Most of the patients with BJI were successfully treated. Failure occurred in 4 patients, which was likely related to a complex interplay of multiple factors — the virulence of the underlying organism (e.g. *Pseudomonas*, *S. aureus*), inadequate source control, shorter duration of induction therapy, and the underlying immune suppression of the host. Not surprisingly, survival was influenced by the hematologic malignancy and for many patients death was due to recurrence or progression of disease (10/26, 38.5%) or another infection (9/26, 34.6%).

Our study has some limitations inherent to its retrospective nature. First, by using ICD codes as our search strategy, we may not have captured all patients diagnosed with BJI. Second, data regarding infections may have been missed if they were not adequately described in the electronic health record. Information outside of the Mayo Clinic such as hospital admissions related to BJI may not have been properly documented or captured. Despite these limitations, our study is the first large cohort that describes the epidemiology of BJI among HSCT recipients. There appears to be a low incidence of BJI following HSCT. Most infections were due to gram-positive organisms, similar to the non-transplant population. Combining surgery and medical therapy is often necessary for treatment success. Survival is often a function of the underlying hematologic disease.

## Figures and Tables

**Table 1:** Baseline Characteristics of Patients in the Study

BASELINE CHARACTERISTIC	n median (range)
DEMOGRAPHICS	
Sex, Female	9
Age (yr)	58 (20-72)
Co-Morbidities	
Diabetes Mellitus	7
Inflammatory/ Rheumatoid Arthritis	4
Autoimmune Disease	4
On Corticosteroid or immune suppressant	24
Chronic Kidney Disease	9
PRIMARY HEMATOLOGIC MALIGNANCY	
Myeloid (AML/MDS/CML)	9
Lymphoid (ALL, CLL, HD, MM, NHL and other lymphomas)	24
TYPE OF TRANSPLANT	
Autologous	19
Allogeneic	14
HEMATOLOGIC TESTS AT TIME OF INFECTION	
Neutropenia (n)	7
Duration of Neutropenia (days)	22 (8-287)
ANC	900 (50-7540)
CHARACTERISTICS OF INFECTION	
Inflammatory markers	
ESR (mm/l h)	46 (1-138)
CRP (mg/L)	38.1 (4.8-346.4)
Synovial Fluid analysis, n (%)	17 (50%)
Cell count (µL)	19,481 (52-350,658)
Positive culture	13/17 (76.5%)
Blood Cultures, n (%) Positive culture	16/25 (64%)
Tissue Cultures, n (%) Positive culture	24/27
CHARACTERISTICS OF TREATMENT	
Choice of Induction Antimicrobial Therapy	
IV Antibiotics alone	24
IV plus oral	8
Oral alone	1
Duration Induction Antimicrobials (days)	34 (3-168)
Duration Maintenance Antimicrobials (days)	29.5(6-287)
OUTCOME	
Treatment Failures	4/33
Reason for Treatment Failure	
Early progression	2
Relapse*	2
Reinfection*	1
Duration from Infection Diagnosis to Treatment Failure (mos)	3.4(0.1-48.5)
Overall Mortality, n (%)	26 (78.8%)
Time to death from infection diagnosis (mos)	13 (0.07-76)
Time to death, successfully treated	13 (0.7-76)
Time to death, treatment failure	39.8 (0.07-70.57)
Cause of Death	
Relapse of Malignancy	10
BJI Infection	1
Other infection	9
Other, non-infectious	1
Unknown	5

* One patient had both relapse and re-infection. LEGEND: AML- Acute myelogenous leukemia; MDS- myelodsyplastic syndrome; CML- chronic myelogenous leukemia; ALL -acute lymphoblastic leukemia; CLL -chronic lymphocytic leukemia; HD - Hodgkin's disease; MM - multiple myeloma; NHL - Non-Hodgkin's lymphoma; ANC - absolute neutrophil count; ESR- erythrocyte sedimentation rate; CRP - C-reactive protein; IV - intravenous.

**Table 2:** Characteristics of Infection by Type.

Type of BJI	N (%)	Male Gender	Age^	Auto HSCT	Time to Infection, (mos)^	ESR/CRP^	Tissue cultures, (n)	Organisms		
								Gram Positive	Gram Negative	Other
Overall	33	24	58	19	39	46/38.1	24/27			
Septic arthritis	15	11	51	10	51	54/72	9/9	MRSA (1), SPN (2), *E. faecalis* (1), CoNS(1), GABHS (1)	KPN (1), *E.cloacae*(1) *P.aeruginosa* (1)	*M. chelonae* (1)
Osteomyelitis	10	8	60	4	30.5	42/26.1	8/10	CoNS (2), MSSA (2), *E. faecalis* (1) *Peptostreptococcus* (2),	KPN (1)	Polymicro-bial (1)
Prosthetic joint infection	6	4	65.5	4	38	51/51	6/6	CoNS (3), MSSA (1), MRSA (1), SPN (1)	None	None
Orthopedic implant infection	2	1	52.5	1	24.5	80.5/8.25	1/2	*C. acnes* (1)	None	None

^Median values FOOTNOTE: MRSA - Methicillin resistant *Staphylococcus aureus*, SPN- *Streptococcus pneumoniae*, CoNS- Coagulase Negative Staphylococcus, GABHS- Grp A Beta Hemolytic Streptococcus, MSSA- Methicillin Susceptible *Staphylococcus aureus*, KPN-*Klebsiella pneumoniae.*

**Table 3:** Description of Treatment

Type of BJI	Treatment Induction, median duration (days)	Surgery, n/N	Medications			Failure (n)	Median time to failure (days)
			Induction	Maintenance	Suppression		
Overall	34	23/33	33	6	8	4	101
SA	29	9/15	CTX (4), VAN (2), LVX (1) DAP (1), NAF (1), CTX+MOX (1) VAN/CEF (1), PCN/GEN (1), VAN/MEM (1), VAN/TZP/CIP (1), CLAR/TOB/TGC (1)	LVX (1), MIN (1)	PCN (2)	1	2
OM	42	6/10	VAN (2), ETP (2), CEF (1), ETP/LVX (1), ETP/VAN (2), CTX/AMP (1), VAN/CIP (1)	MOX (1)	PCN (2), PCN/MIN (1), AMOX/TMP-SFZ (1)	0	NA
PJI	47	6/6	CFZ (1), VAN (2), VAN/RIF (2), VAN/MOX(1)	TMP/SFZ (1)	TMP/SFZ (1), MIN (1)	3	191
OII	21.5	2/2	AMOX/CLAV (1), DAP (1)	CEX (1), DOX (1)	NONE	0	NA

FOOTNOTE: SA - Septic arthritis, OM - Osteomyelitis, PJI -Prosthetic joint infection, OII- orthopedic implant infection, CTX - Ceftriaxone, VAN- Vancomycin, LVX - Levofloxacin, DAP- Daptomycin, NAF- Nafcillin, MOX- Moxifloxacin, CEF- Cefepime, PCN- Penicillin, GEN- Gentamicin, MEM- Meropenem, TZP- Piperacillin-tazobactam, CIP- Ciprofloxacin, CLAR- Clarithromycin, TOB -Tobramycin, TGC- Tigecycline, ETP- Ertapenem, AMP - Ampicillin, CFZ - Cefazolin, RIF - Rifampin, AMOX/CLAV- Amoxicillin-Clavulanate, MIN- Minocycline, TMP/SFZ - Trimethoprim-sulfamethoxazole, CEX -Cephalexin, DOX- Doxycycline, AMOX- Amoxicillin, NA- not applicable
